# Pulmonary gas exchange evaluated by machine learning: a computer simulation

**DOI:** 10.1007/s10877-022-00879-1

**Published:** 2022-06-13

**Authors:** Thomas J. Morgan, Adrian N. Langley, Robin D. C. Barrett, Christopher M. Anstey

**Affiliations:** 1 Mater Research, Mater Health Services and University of Queensland, Stanley Street, South Brisbane, Brisbane, QLD 4101 Australia; 2 Intensive Care Department, Mater Health Services, Stanley Street, South Brisbane, Brisbane, QLD 4101 Australia; 3 University of Queensland, Brisbane, QLD 4072 Australia; 4 Brisbane, QLD Australia; 5 Griffith University, Gold Coast, QLD 4215 Australia

**Keywords:** Computer simulation, Gas exchange, Lung model, Machine learning, MIGET format

## Abstract

Using computer simulation we investigated whether machine learning (ML) analysis of selected ICU monitoring data can quantify pulmonary gas exchange in multi-compartment format. A 21 compartment ventilation/perfusion (V/Q) model of pulmonary blood flow processed 34,551 combinations of cardiac output, hemoglobin concentration, standard P50, base excess, VO_2_ and VCO_2_ plus three model-defining parameters: shunt, log SD and mean V/Q. From these inputs the model produced paired arterial blood gases, first with the inspired O_2_ fraction (FiO_2_) adjusted to arterial saturation (SaO_2_) = 0.90, and second with FiO_2_ increased by 0.1. ‘Stacked regressor’ ML ensembles were trained/validated on 90% of this dataset. The remainder with shunt, log SD, and mean ‘held back’ formed the test-set. ‘Two-Point’ ML estimates of shunt, log SD and mean utilized data from both FiO_2_ settings. ‘Single-Point’ estimates used only data from SaO_2_ = 0.90. From 3454 test gas exchange scenarios, two-point shunt, log SD and mean estimates produced linear regression models versus true values with slopes ~ 1.00, intercepts ~ 0.00 and R^2^ ~ 1.00. Kernel density and Bland–Altman plots confirmed close agreement. Single-point estimates were less accurate: R^2^ = 0.77–0.89, slope = 0.991–0.993, intercept = 0.009–0.334. ML applications using blood gas, indirect calorimetry, and cardiac output data can quantify pulmonary gas exchange in terms describing a 20 compartment V/Q model of pulmonary blood flow. High fidelity reports require data from two FiO_2_ settings.

**Supplementary Information:**

The online version contains supplementary material available at 10.1007/s10877-022-00879-1.

## Introduction

More than 50 years ago John West published his landmark model of pulmonary gas exchange [1], building on the work of predecessors [2]. The model is characterised by volumes of inspired gas (V) and mixed venous blood (Q) equilibrating in 10 to 100 virtual lung compartments governed by log normal distributions of alveolar ventilation and pulmonary capillary blood flow across compartmental V/Q ratios [1, 3, 4].

The multiple inert gas technique (MIGET), an investigative tool based on West’s model [5, 6], has provided mechanistic detail on impaired gas exchange. MIGET evaluations are technically challenging procedures in which six inert gases spanning a range of solubilities are infused in saline until equilibration. Plots of pulmonary retention and excretion versus gas solubility are constructed from gas chromatographic measurements and ‘transformed’ respectively into distributions of blood flow and ventilation against a logarithmic scale of V/Q ratios spread across 50 compartments [5, 7].

MIGET has identified shunt (V/Q = 0) as the dominant cause of hypoxaemia in the acute respiratory distress syndrome (ARDS) and lobar pneumonia, whereas in chronic obstructive pulmonary disease (COPD) and in some patients with COVID-19 pneumonia hypoxaemia is primarily from mixed venous equilibration in low V/Q compartments [8–10]. Bimodal distributions have been observed in patients with COPD, asthma [3] and ARDS [11].

Despite its ‘gold standard’ status, the complexity of MIGET has obliged clinicians to track pulmonary gas exchange via alternative indices, usually those categorized as ‘tension’ or ‘content’-based [12]. Venous admixture (VA) is the classic content-based index [13], while tension-based indices include the A–a gradient, used in APACHE risk algorithms [14], and the ratio between the arterial oxygen tension and the inspired oxygen fraction (PaO_2_/FiO_2_ ratio or PF ratio), important in ARDS diagnosis and stratification [15].

These indices show significant signal variability [16], but their greatest drawback is the limited information provided on the underlying pulmonary pathophysiology. The VA approach of Riley and Cournand [13, 17] is more informative on this aspect, but hampered by inherent over-simplification. This is because VA (V/Q = 0) is one of just two perfused compartments (V/Q = 0 and 1). All oxygen transfer deficits are corralled within VA, in other words as true shunt, leaving no ability to tease out contributions from low V/Q compartments. For clinicians this can be a crucial distinction, for example in managing COVID-19 pneumonia (see “Discussion” section) [10]. Similarly, the effects of high V/Q are incorporated in a single dead space estimate (V/Q = ∞). As a final drawback, accurate VA calculations require mixed venous blood for analysis [12].

In part to address these shortcomings, scaled back variations on the MIGET framework have been proposed [18–21]. Prominent among these is the automatic lung parameter estimator (ALPE) [18], described as a ‘simple bedside alternative to MIGET’. ALPE has been shown to match complex MIGET calculations in experimental lung injury [22, 23], and is now finding application in clinical research [24] and as the key component of a commercial package (www.mermaidcare.com) designed for monitoring and decision support.

Like MIGET, shunt is given conventionally in ALPE assessments as percentage of cardiac output. However, unlike MIGET, ALPE models ‘low’ and ‘high’ V/Q mismatch as partial pressure differentials (to be distinguished from diffusion limitation) across imposed ‘partitions’ between blood and alveolar gas. Specifically, ‘low’ V/Q mismatch is represented by the fall in PO_2_ from alveolar gas to pulmonary end-capillary blood, and ‘high’ V/Q mismatch as the rise in PCO_2_ across the same interface.

We suggest that machine learning (ML) could add value in this ‘scaled back MIGET’ space [25, 26]. With data inputs close to those used by ALPE it should be possible for trained ML applications to generate detailed pulmonary assessments. These could take the form of a shunt estimate plus separate parameters defining log normal distributions of blood flow across compartmental V/Q ratios. Critical care physicians would then be provided with prompt actionable diagnostic information presented in a familiar format. Added bonuses could include shorter measurement intervals with a reduced requirement for FiO_2_ ‘switching’ (at present ALPE requires up to four FiO_2_ ‘switches’).

To investigate this possibility, we tested the following hypotheses in silico:

1. Trained ML applications using data normally sourced from blood gas analysis, indirect calorimetry, and cardiac output measurements can quantify pulmonary gas exchange in terms describing a multi-compartment V/Q model of pulmonary blood flow.
2. Consistent ML reports require measurement data at no more than two FiO_2_ settings.

## Materials and methods

**Fig. 1:**
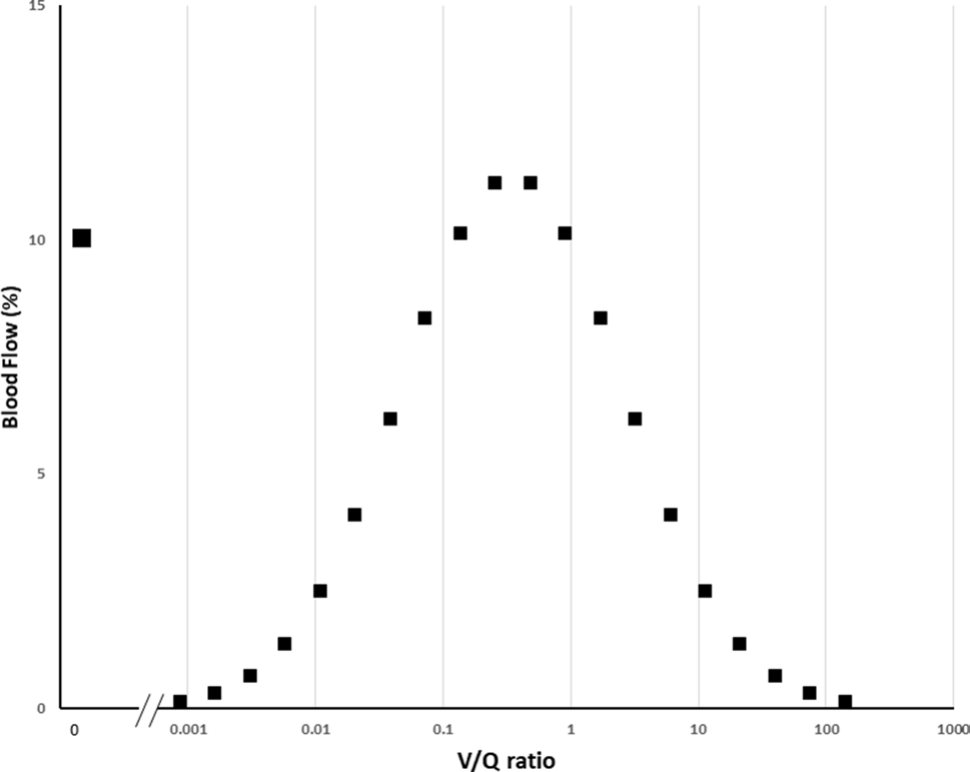
Graphical illustration of modelled blood flow through 20 gas exchanging compartments plus a single shunt compartment (V/Q = 0). Shunt is set at 10% of total pulmonary blood flow. Note the log normal distribution of the non-shunt pulmonary blood flow according to compartment V/Q ratios. In this example log SD = 2.0 and flow distributional V/Q mean = 0.35

To test the above hypotheses, we exposed selected ML applications to simulated clinical monitoring data routinely available from blood gas analysis, indirect calorimetry, and cardiac output measurements. Scenarios were constructed with these data to represent a diverse mix of O_2_ consumption (VO_2_) and delivery, CO_2_ production (VCO_2_) and transport, hemoglobin-oxygen affinity, and respiratory and metabolic acid–base status. Paired blood gases were generated in each simulation by a 21-compartment model of pulmonary blood flow governed by three input values: shunt percentage, log standard deviation (log SD) and distributional mean (Fig. 1, for more model detail and core equations, see Supplementary Material).

To make the evaluation, ML applications trained on this material were challenged with simulated monitoring data from ‘unseen’ test scenarios, the goal in each case being to back-generate the three governing model parameters of pulmonary blood flow distribution (shunt, log SD and mean). These estimates were then compared with ‘true’ model input values for the same scenarios.

Steps in this process were as follows:

1. Arterial blood gases were produced by the lung model at two structured settings of inspired oxygen fraction (FiO_2_) (see below) in response to unique input combinations of the three parameters defining model pulmonary blood flow distribution (shunt, log SD and mean, Table 1) plus one value from each of six monitoring categories (Table 2) available from blood gas analysis, indirect calorimetry, and cardiac output measurements.

**Table 1:** Model defining parameters

Variable	Range
Shunt (% of pulmonary blood flow)	5.5 to 36.6
Log SD	0.27 to 2.20
Mean V/Q	0.089 to 1.7

*V* volume of inspired gas, *Q* volume of mixed venous blood, *SD* standard deviation

**Table 2:** Monitoring inputs with ranges

Variable	Range
VCO_2_ (mL/min)	190 to 225
VO_2_ (mL/min)	189 to 375
Hemoglobin (G/dL)	6.0 to 17.5
P50st (mmHg)	20.0 to 32.8
Base excess (mEq/L)	− 9 to + 10
CO (L/min)	4.2 to 6.5

*VCO*_*2*_ total carbon dioxide production rate, *VO*_*2*_ total oxygen consumption rate, *P50st* standard P50, *CO* cardiac output
2. Using a Python program, 34,551 unique input combinations were built around a core set of 7500.
3. Model calculations were run from VBA sub-routines (Excel, Microsoft, Redmond, WA) until stable outputs were achieved for pH, PCO_2_, PO_2_ and Hb saturation in arterial and mixed venous blood and in the pulmonary end-capillary blood of each of the 20 non-shunt compartments.
4. For each input combination, the FiO_2_ generating an arterial oxygen saturation (SaO_2_) of 0.90 was determined by iteration, ensuring that in each case 0.21 ≤ FiO_2_ ≤ 0.90.
5. On attainment of SaO_2_ = 0.90, values were logged for FiO_2_, arterial pH, arterial PO_2_ (PaO_2_), arterial PCO_2_ (PaCO_2_), calculated PF ratio and calculated venous admixture (VA).
6. For the second calculation the FiO_2_ was increased by 0.1 and the model run again.
7. Values for SaO_2_ and calculations of VA, and PF ratios were logged at this higher FiO_2_.
8. With data from SaO_2_ = 0.90 as baseline, changes at the higher FiO_2_ in SaO_2_ (Dsat), VA (DVA) and PF ratios (DPF) were calculated and logged.
9. This sequence performed 34,551 times generated the final dataset.

### ML analysis of completed dataset

1. After pre-processing to reduce redundancies, data rows were formatted as in Table 3 and subjected to randomization.

**Table 3:** Example of pre-processed data for ML training

Shunt	Log SD	Mean	FiO_2_	CO_2_load	O_2_pull	pH	PaCO_2_	PaO_2_	P50st	VA	BE	Hb	DVA	Dsat	DPF
24	2	0.8	0.69	42.9	55.0	7.55	24.9	48.5	26.0	32.6	0.0	10.8	− 1.2	0.02	− 1.87
24	2	0.6	0.77	42.9	55.0	7.46	33.1	53.2	26.0	33.6	0.0	10.8	− 1.2	0.02	− 0.86
24	2	0.7	0.72	42.9	55.0	7.51	28.4	50.5	26.0	33.1	0.0	10.8	− 1.2	0.02	− 1.45
20	2	0.3	0.88	42.9	55.0	7.39	59.0	69	31.0	34.4	8.0	10.8	− 1.9	0.03	2.55
15	2	0.3	0.75	42.9	55.0	7.4	52.7	68	31.0	32.3	6.0	10.8	− 1.9	0.03	0.69
24	2	0.6	0.76	42.9	55.0	7.46	33.1	53.1	26.0	33.6	0.0	10.8	− 1.2	0.02	− 0.90
20	2	0.3	0.87	42.9	55.0	7.39	59.0	59.2	26.6	34.5	8.0	10.8	− 1.9	0.03	2.11
20	2	0.3	0.87	42.9	55.0	7.39	58.9	48.9	22.0	34.6	8.0	10.8	− 1.9	0.03	1.70
15	2	0.3	0.74	42.9	55.0	7.4	52.6	58.3	26.6	32.4	6.0	10.8	− 1.9	0.03	0.45
15	2	0.3	0.73	42.9	55.0	7.4	52.6	46	21.0	32.5	6.0	10.8	− 1.8	0.03	0.22

*CO*_*2*_*load* VCO_2_/CO, *O*_*2*_*pull* VO_2_/CO, *VA* venous admixture (%), *BE* base excess (mEq/L), *Hb* blood hemoglobin concentration (G/dL), *DVA* delta venous admixture (%), *Dsat* delta arterial saturation, *DPF* delta PF ratio

(2) The randomized dataset was partitioned into sequential split fractions (70%:20%:10%) for ML training, validation and testing respectively.
(3) The test fraction was subjected to trained ML analysis with columns containing the model-defining values of pulmonary blood flow (shunt, log SD and mean) ‘held back’ to allow blinded estimates.
(4) Two categories of ML estimates were performed:
  - ‘Single-Point’ estimates were derived by ML analysis of 10 variables confined to model input and output logs for SaO_2_ = 0.90. Input variables were ‘CO_2_load’, ‘O_2_pull’, standard P50 (P50st) [27], base excess, BE [28], and blood haemoglobin concentration (Hb). Output variables were FiO_2_, arterial pH, PaCO_2_, PaO_2_, and VA (Table 3).
(b) ‘Two-Point’ estimates were derived after inclusion of three additional variables consisting of DVA, Dsat and DPF (Table 3), all obtained from model output logs following the 0.10 FiO_2_ increment.

### ML methodology

We used open-source ML algorithms implementing linear regression techniques [Supplementary Material Table 1(s)]. It became evident during the validation process that multiple simultaneous models in a ‘stacked’ or ‘ensemble’ configuration outperformed any single model. The stacking process used simple linear regression at the output layer to combine the contributions from individual models.

Model stacks were tested using ‘StackingRegressor’ from the ‘sklearn’ Python library (https://scikit-learn.org/stable/). Models were trained using correlation (‘R’ and ‘R^2^), mean absolute error (‘MAE’) and by comparing the slope and distance from zero intersection of the line of best fit.

See ‘Supplementary Material’ for more detail of ML methodologies employed.

### Statistical analysis

Prior to analysis, the comparison data were checked for completeness, accuracy, and consistency.

Two-way (univariate) comparisons were made using standard linear regression. Post-estimation diagnostics were run on all models. Due to the large size of the dataset, these included checking model residuals for normality, using both the Kolmogorov–Smirnov test and a normal probability plot and heteroskedasticity, using the Breusch–Pagan and Cook–Weisberg tests. For each predictor, the regression slope (β) and its p-value were tabulated along with the equation intercept and the overall R^2^ value.

Kernel density plots and graphical Bland and Altman analyses [29] were constructed to enable visual comparisons of single-point and two-point results for each variable (shunt, log SD, and mean estimates) versus the true values.

STATA_TM_ (v17.0) was used for all analyses with the level of significance set throughout at α < 0.05.

## Results

From the final dataset of 34,551 data rows, 31,097 rows were allocated for ML training and validation and the remaining 3454 rows for testing.

**Fig. 2:**
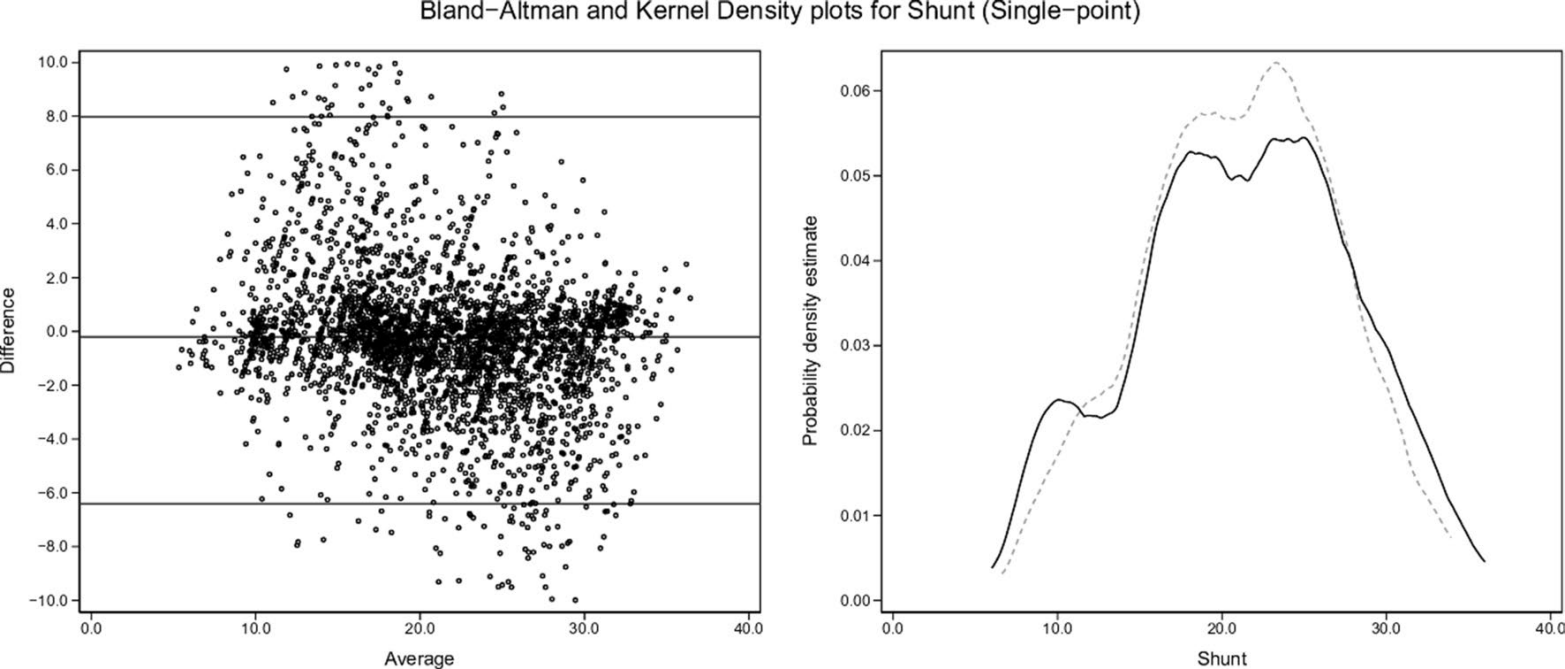
Shunt (single-point). Two subplots are illustrated. The Bland–Altman (BA) plot illustrates the 3454 points. For clarity, each point is horizontally jittered by ± 1% of the value of the independent variable. Horizontal plot lines indicate the median and 95% confidence interval for the difference (enumerated in Table 5). The kernel density estimate (KDE) plot illustrates the distribution of observations for the independent variable. The solid line is the true value of the variable with the dashed line indicating the modeled variable. Each subplot shares the same X-axis scale. Both X-axis units and the Y-axis units in the BA plot are defined by the independent variable. The Y-axis in the KDE plot is dimensionless

**Fig. 3:**
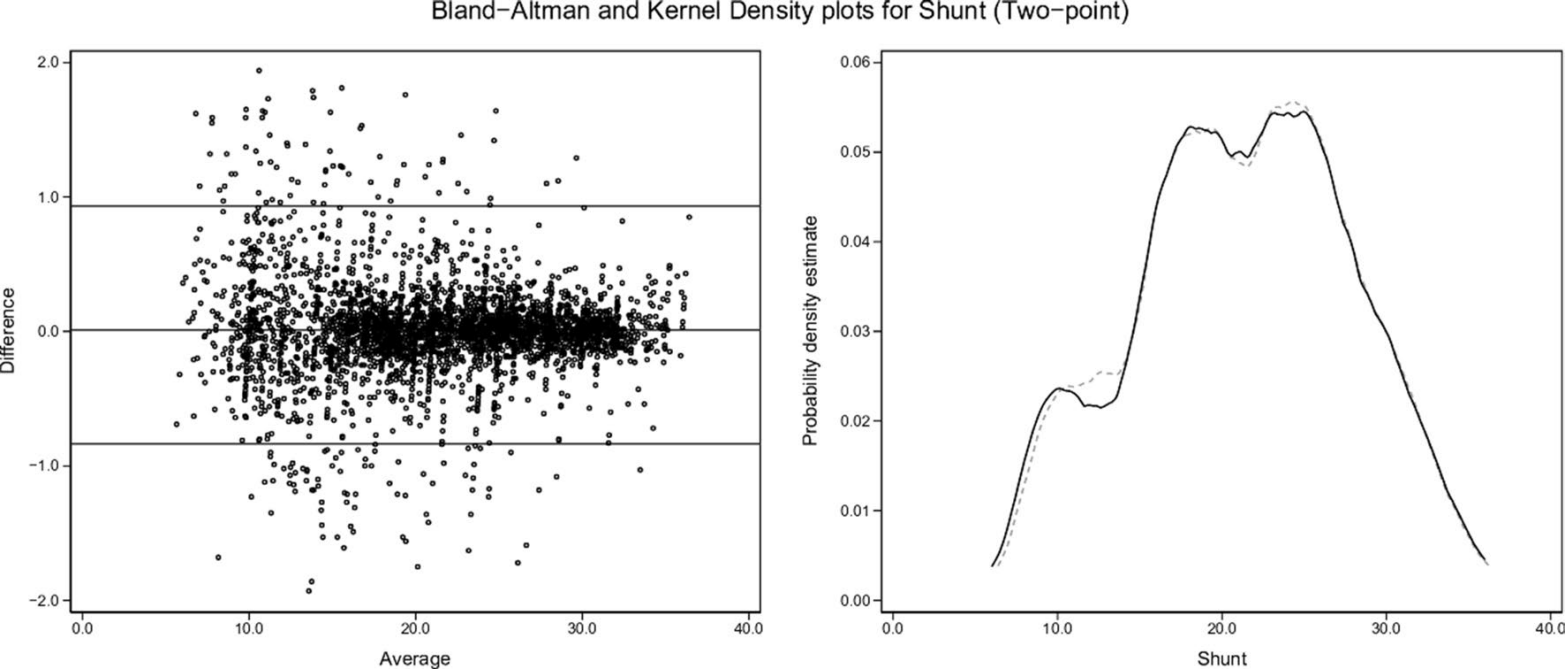
Shunt (two-point). Description as for Fig. 2

**Fig. 4:**
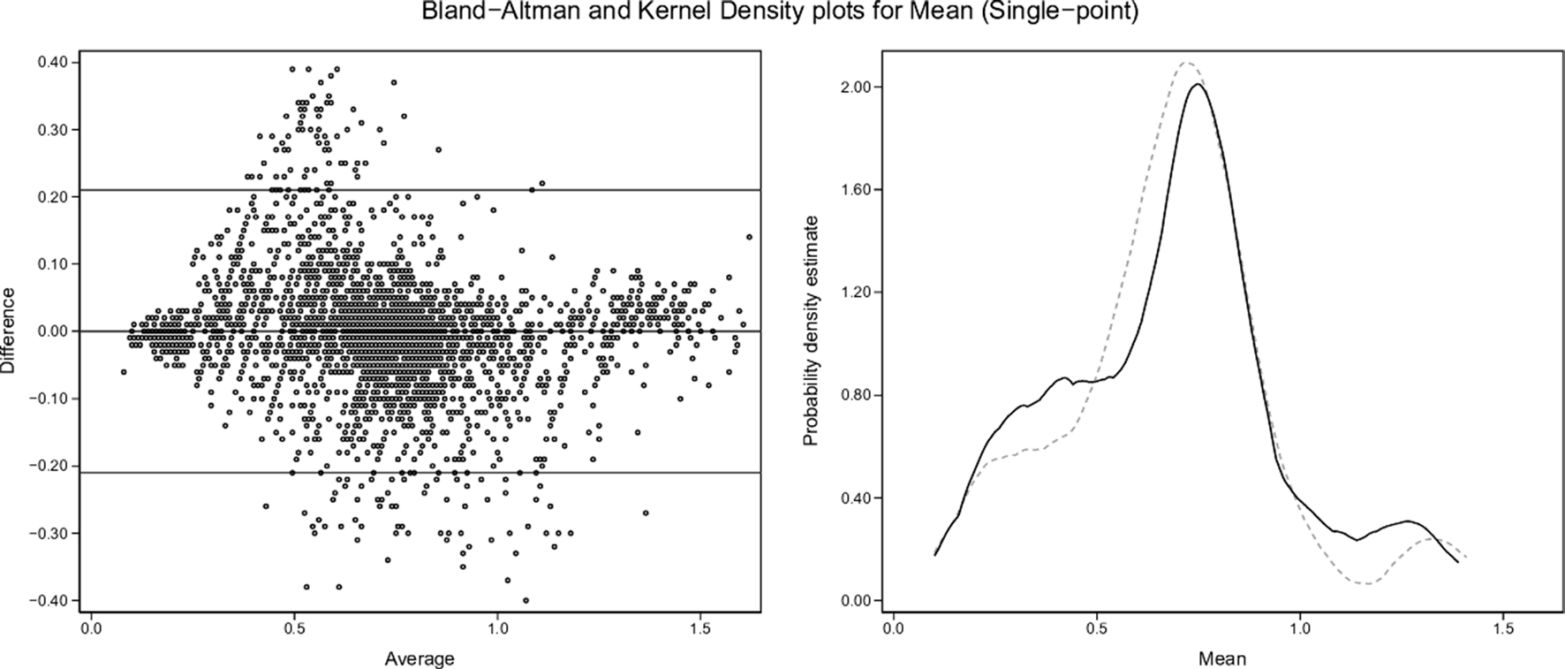
Mean (single-point). Description as for Fig. 2

**Fig. 5:**
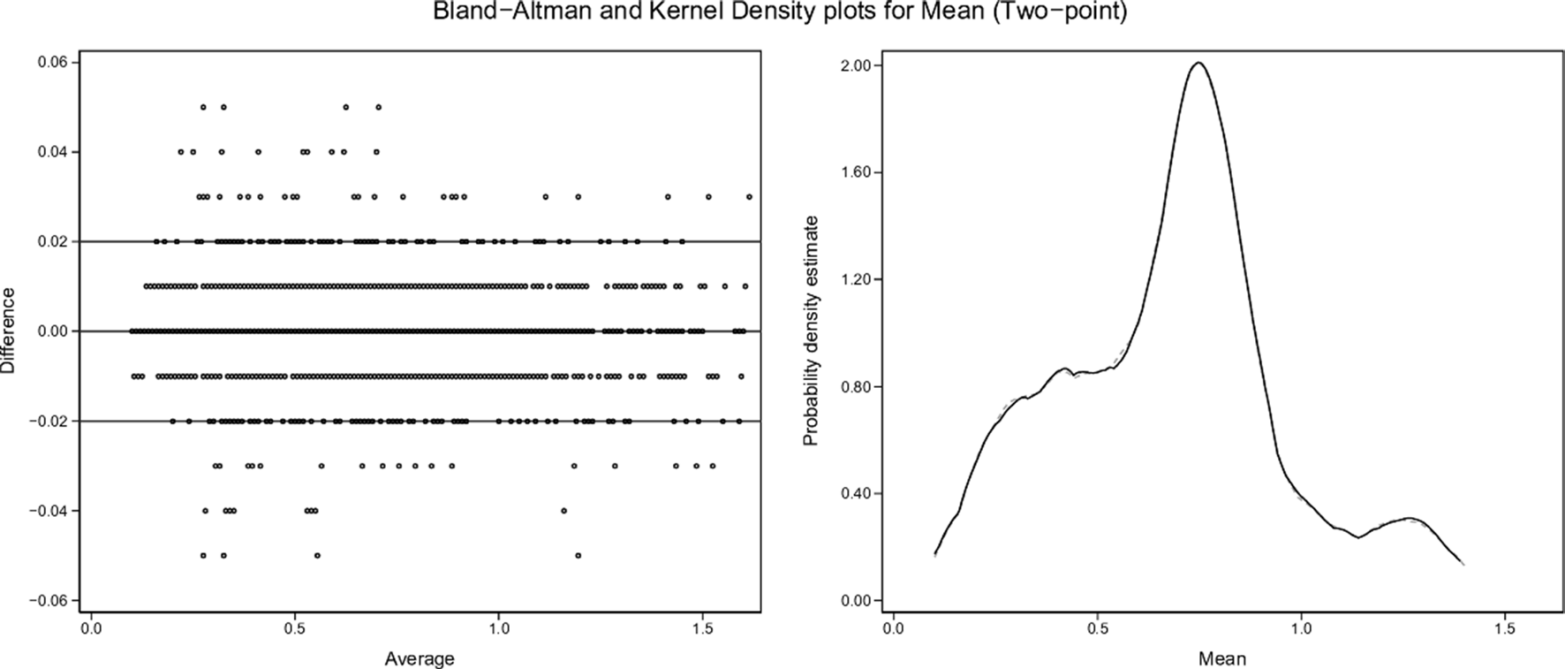
Mean (two-point). Description as for Fig. 2

**Fig. 6:**
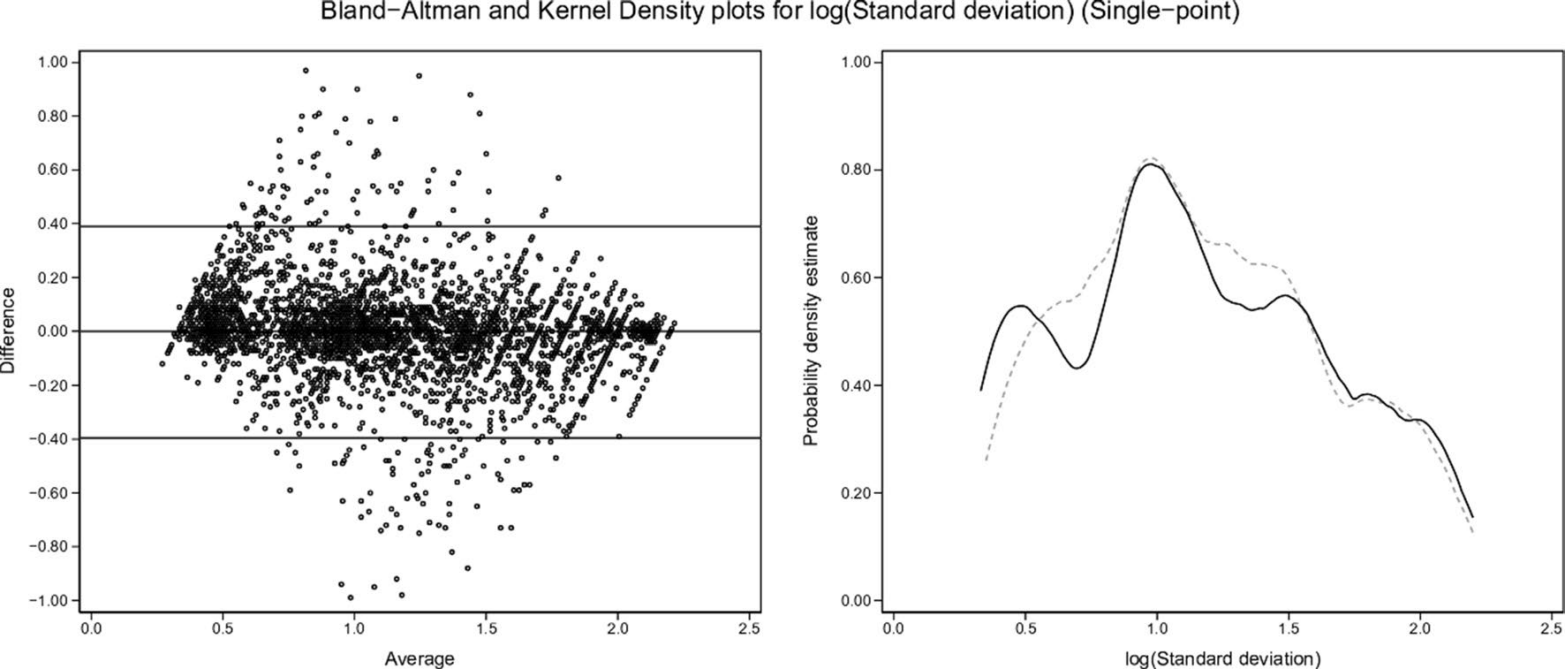
Log SD (single-point). Description as for Fig. 2

**Fig. 7:**
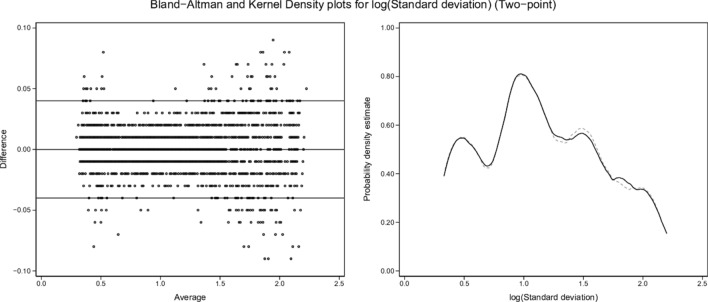
Log SD (two-point). Description as for Fig. 2

From the 3454-row test-set, kernel density and Bland and Altman plots of single-point and two-point estimates by ML versus true values of shunt, log SD and mean are set out in Figs. 2, 3, 4, 5, 6 and 7. All distributions are non-normal. Corresponding regression data are reported in Table 4, and Bland and Altman data in Table 5.

**Table 4:** Linear regression analysis: single-point and two-point estimates of shunt, log SD and mean versus true input values

	Shunt	Log SD	Mean
Single-point	R^2^ = 0.77 β = + 0.991 (p < 0.001) Constant = + 0.334	R^2^ = 0.87 β = + 0.993 (p < 0.001) Constant = + 0.047	R^2^ = 0.89 β = + 0.993 (p < 0.001) Constant = + 0.009
Two-point	R^2^ > 0.99 β = + 1.001 (p < 0.001) Constant = − 0.038	R^2^ > 0.99 β = + 1.000 (p < 0.001) Constant = − 0.001	R^2^ > 0.99 β = + 1.001 (p < 0.001) Constant = − 0.001

**Table 5:** Results for Bland–Altman plots

	Median	95% CI
Single-point		
Shunt	− 0.21	− 6.42, + 7.97
Mean	0.00	− 0.21, + 0.21
SD	0.00	− 0.40, + 0.39
Two-point		
Shunt	+ 0.01	− 0.84, + 0.93
Mean	0.00	− 0.02, + 0.02
SD	0.00	− 0.04, + 0.04

95% CI estimates were calculated as the 2.5 and 97.5 percentiles around the median [43]

### Two-point estimates

Two-point estimates of shunt, log SD and mean produced regression models with almost identical results (Table 4), with β ~ 1.00, intercept ~ 0.00 and R^2^ ~ 1.00 for each of the test-set variables. The kernel density and Bland and Altman plots confirmed close agreement with true values (Figs. 3, 5, 7; Table 5).

### Single-point estimates

From Figs. 2, 4 and 6 and Tables 4 and 5, single-point estimates showed close concordance but less consistent reflections of true values. Ranges from the regression models of the three estimate categories versus true values were R^2^ = 0.77–0.89, β = 0.991–0.993, and intercepts = 0.009–0.334 (Table 4).

## Discussion

Using computer simulation, we found that blinded ML analysis of monitoring data replicating diverse gas exchange scenarios, including blood gases generated by a 21-compartment V/Q model of pulmonary blood flow, could back-generate the model’s governing parameters. This was achieved with ‘stacked regressor’ ML ensembles trained and tested on blood gas, indirect calorimetry, and cardiac output data over a broad spectrum of gas exchange equilibria. In each simulation ML accurately delineated pulmonary blood flow as shunt percentage plus the key descriptors (log SD and mean) of log normal flow distributions to gas exchanging compartments according to their V/Q ratios. This is essentially pulmonary blood flow in MIGET format.

Measurements adopted for the simulation are available from current ICU monitoring devices [30]. Point of care blood gas analysis has been routine in ICU practice for decades. Indirect calorimetry is now recommended as a nutritional guide for critically ill mechanically ventilated patients [31–33]. Low invasive cardiac output monitoring, although not without problems [34–36], is mainstream in contemporary ICUs. The application of artificial intelligence in critical illness monitoring and decision support is itself no longer a novel concept [26].

The dataset to train, validate and test the ML applications was derived from systematically varied input combinations of the three model defining parameters (shunt, log SD, and mean, Table 1), linked to four direct measurements (cardiac output, VO_2_, VCO_2_, and Hb; Table 2) and two calculated parameters (BE, P50st; Table 2). To complete each scenario the model generated paired sets of arterial blood gases in response to these inputs at two structured FiO_2_ settings. The final dataset represented approximately 35,000 unique scenarios covering a diverse mix of O_2_ delivery and consumption, CO_2_ production and transport, hemoglobin-oxygen affinity, and respiratory and metabolic acid–base status.

ML was then able to back-generate the model-defining parameters of 3454 test scenarios in blinded fashion using only the blood gas measurements along with inherent derived values (BE, P50st, VA, PF ratios) plus cardiac output, VO_2_, VCO_2_, and the baseline FiO_2_. ML estimates from single-point data (recorded at baseline SaO_2_ = 0.90) showed sufficient concordance with true values to reflect trends in all three key model parameters. However, a second equilibration introduced a dynamic component, captured by ML via changes in VA (DVA), PF ratios (DPF) and saturation (Dsat). This two-point approach enabled high fidelity identification of all three key model descriptors (Figs. 3, 5, 7; Tables 4, 5).

The simulation was designed to emulate a practical two-step procedure in which arterial blood gas analysis with oximetry is performed with the FiO_2_ adjusted for SaO_2_ = 0.90 (using SpO_2_ as initial guide). This is followed by a second set of blood gases after increasing the FiO_2_ by 0.10. During this process, once only measurements of cardiac output, VO_2_ and VCO_2_ are also recorded. ML then quantifies the defining parameters of the diagnostic model(s) of choice from relationships embedded in the data.

It should be possible to train ML applications in other diagnostic models such as the ALPE system, which like the approach considered here devolves to three key parameters [18, 37], in that case shunt and partial pressure gradients across modelled blood/gas ‘partitions’ representing ‘high V/Q’ and ‘low V/Q’ mismatch. It is also conceivable that larger training datasets with wider input ranges could enable accurate single-point ML reports from data ‘snapshots’ collected at any working FiO_2_. One further possibility for future investigation is that training sets formatted to target specific model variants, for example bimodal flow distributions [38], could extend ML reporting to these complexities.

Informative ‘on the spot’ gas exchange evaluations can facilitate management decisions, as mentioned in the Introduction. A contemporary example might be a ventilated patient with pneumonia and hypoxemia with a PF ratio < 100. To decide on a safe course of action clinicians should be able to distinguish between two extremes of lung pathophysiology. At one extreme the disturbed oxygenation represents a large right to left shunt in the context of low pulmonary gas volumes, typical of recruitable ARDS. At the other pulmonary gas volumes are normal and shunt is minimal, the hypoxemia arising instead from widespread low V/Q ratios due to maldistributed lung perfusion, a situation more characteristic of COVID-19 with multiple pulmonary vascular thrombi. In the latter circumstance, recruitment maneuvers and major manipulations of positive end expiratory pressure (PEEP) would be contraindicated [10, 38]. Varying combinations of the two extremes complete the spectrum of possibilities.

Based on our simulation, ML evaluations could make these distinctions rapidly without a need for specialized imaging. Equivalent diagnostic assessment by the current ALPE system would take 10 to 15 min, involve up to four FiO_2_ ‘switches’, and report VQ mismatch as partial pressure gradients [24, 37].

### Some caveats

The model of pulmonary blood flow used to generate the blood gases follows the basic West model format. Several modifications and simplifications were employed. These are detailed in the Supplementary Material.

The simulation assumes error-free measurements, whereas some degree of error is intrinsic to measurements of cardiac output [36], indirect calorimetry [39], and the measured and derived elements of blood gas analysis [12]. Indirect calorimetry has increased error potential at FiO_2_ ≥ 0.7 or PEEP > 12, both encountered in severe respiratory failure [39]. Other risk factors include circuit leaks, bronchopleural fistulae, and possibly extracorporeal circulations.

We have not attempted a sensitivity analysis. However, it is noteworthy that ALPE, an advanced system now in service, is subject to similar error susceptibilities. ALPE evaluations require a single arterial blood gas analysis and one cardiac output measurement or estimate, along with measurements at three to five different FiO_2_ settings of VO_2_, VCO_2_, arterial oxygen saturation by pulse oximetry (SpO_2_), and end-tidal O_2_ and CO_2_ fractions [24]. Despite measurement intervals of 10–15 min with up to four FiO_2_ ‘switches’, any signal distortion from absorption atelectasis [40] and altered hypoxic pulmonary vasoconstriction [41] is regarded as minor [37].

Further, the MIGET gold-standard itself relies on a series of measurements and techniques all prone to error, including but not limited to cardiac output and minute ventilation measurements, collection of mixed expired gas without condensation-induced loss of dissolved gases, and gas chromatographic concentration measurements of six inert gases in both mixed expired gas and the gas phases above blood samples [4].

The low baseline arterial saturation (SaO_2_ = 0.90) was selected to allow a subsequent 0.10 FiO_2_ step-up within the bounds of FiO_2_ ≤ 1.00. Although SaO_2_ = 0.90 is at the hypoxemia threshold [12], it is considered adequate for tissue oxygenation in the absence of anemia and low cardiac output, albeit with limited supportive evidence [42]. Of historical interest, older versions of the automated ALPE system could manipulate baseline SaO_2_ to values as low as 0.85, if necessary using FiO_2_ < 0.21 [18].

Dataset shunt, log SD and mean values retained uneven distributions across their respective ranges, as illustrated by the test-set kernel density plots (Figs. 2, 3, 4, 5, 6, 7). Greater training set uniformity may have produced more consistent single-point estimations. Barriers to uniformity included the automatic rejection of input combinations in which SaO_2_ ≠ 0.90 when FiO_2_ ≥ 0.21 ≤ 0.90.

## Conclusions

We conclude based on computer simulations of diverse gas exchange scenarios that trained ML applications using data sourced from blood gas analysis, indirect calorimetry, and cardiac output measurements can quantify pulmonary gas exchange in terms used to describe multi-compartment V/Q models of pulmonary blood flow. High fidelity ML reports require measurement data at no more than two FiO_2_ settings, subject to measurement accuracy.

## Supplementary Information

Below is the link to the electronic supplementary material.

Supplementary file1 (DOCX 174 kb)
