# Supplementary material for: Pulmonary gas exchange evaluated by machine learning: a computer simulation
Source: J Clin Monit Comput. 2022 Jun 13;37(1):201–10. doi: 10.1007/s10877-022-00879-1 (PMC9188913; doi:10.1007/s10877-022-00879-1)
Supplement: Supplementary file 1 — Supplementary file1 (DOCX 174 kb) [file 10877_2022_879_MOESM1_ESM.docx]

Supplementary Material

The model

A model of pulmonary gas exchange was configured to generate paired arterial blood gas data at structured FiO_2_ settings according to nine constraints, consisting of three governing parameters of pulmonary blood flow distribution (shunt, log SD and mean) and six selected ‘bedside’ monitoring inputs (Tables 1 and 2, main article). The model, run from VBA sub-routines (Excel, Microsoft, Redmond, WA) is based on the approach of West [1] with certain simplifications and methodological variations. The following are its key characteristics:

1. One shunt compartment (V/Q = 0) and 20 gas exchanging compartments.
2. Modelling centered on pulmonary blood flow distribution.
3. Non-shunt pulmonary blood flow distributed (by sub-routine) on a log normal basis across a spread of compartmental V/Q ratios as determined by two parameters: mean V/Q and log standard deviation (log SD).
4. Pulmonary blood flow distribution is thus defined in full by three parameters: shunt, log SD and mean.
5. Compartmental ventilation is a dependent variable derived from compartmental blood flow and its allocated V/Q ratio. Distribution of V versus V/Q ratios is log normal, with the same log SD as Q but a separate mean which is also dependent on and defined by blood flow parameters [Figures 1 (s) and 2 (s)].
6. As with the West approach, diffusion limitation is not modelled directly.
7. Also in line with the original West model, blood flow and ventilation in healthy lungs have narrow distributions with means centred near V/Q = 1 [(Figure 1(s)].
8. V/Q heterogeneity expands as log standard deviations increase and the two means diverge [Figure 2(s)]. Greater proportions of mixed venous blood then equilibrate in low V/Q compartments causing pulmonary capillary oxygen uptake to fall, culminating in zero uptake in pure shunt (V/Q = 0).
9. At the same time CO_2_ elimination becomes inefficient as more alveolar ventilation is directed to higher V/Q compartments.


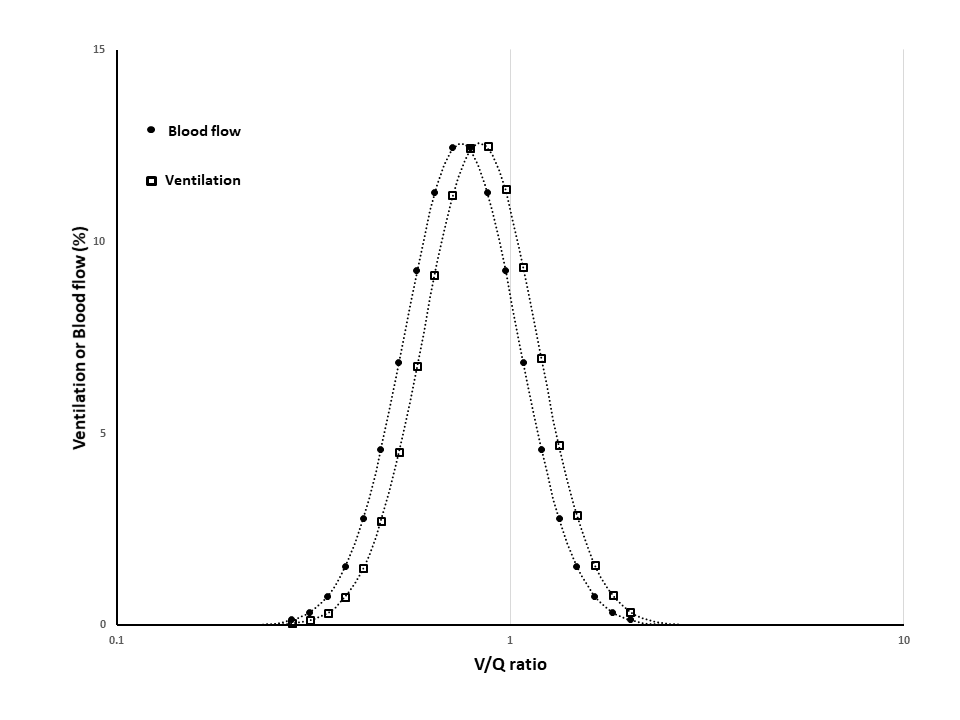


Figure 1 (s). Modelled blood flow and corresponding ventilation distributions in healthy lungs according to V/Q ratios in 20 gas exchanging compartments. Both distributions are log normal. Parameters for blood flow: Shunt 0%, log SD = 0.33, V/Q distributional mean = 0.75. Ventilation: log SD = 0.33, V/Q distributional mean = 0.84. In healthy lungs blood flow and ventilation distributions are narrow and close to unimodal.


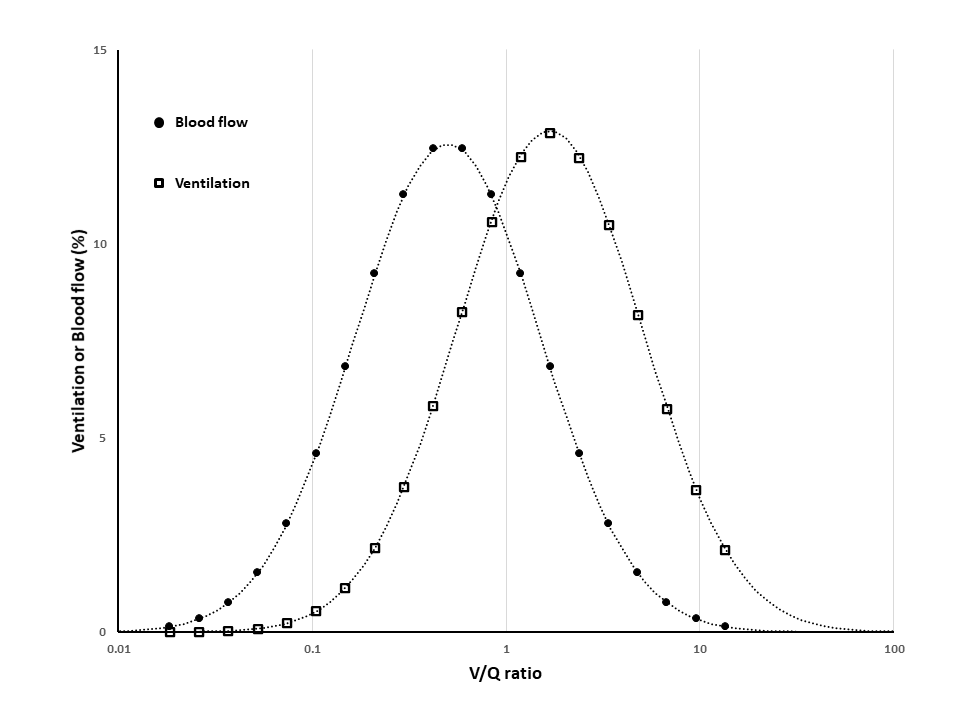


Figure 2 (s). Modelled blood flow distribution and corresponding ventilation in lungs with significant V/Q mismatch. Parameters for blood flow: Shunt 0%, log SD = 1.1, V/Q distributional mean = 0.5. Ventilation: log SD = 1.1, V/Q distributional mean = 1.65. Note the increased V/Q heterogeneity which accompanies expanding blood flow and ventilation distributions, with both means diverging.

Comparisons with the original West model:

1. We elected not to incorporate nitrogen exchange, which impacts gas transfer mainly at very low V/Q ratios [1].
2. Blood CO_2_ content (CbCO_2_) is calculated by applying the Douglas equation [2] (Core Equations 2 and 3) rather than via the Kelman sub-routine used by West [3].The Douglas equation is a 1988 update of the ‘McHardy – Visser’ equation [4], with new constants derived from experiments on volunteers [2]. Agreement with these experimental data was found to be closer than with the relevant Kelman sub-routine [3].
3. We modelled hemoglobin – oxygen dissociation using a version of Siggaard-Andersen’s tan H equation [5] (Core Equation 8), again in preference to the earlier Kelman sub-routine [6]. The tan H method is well-established and maintains the closest agreement with the standard curve if SO_2_ ≤ 0.97.
4. We applied a fixed BE Haldane coefficient of 0.22 [7] (Core Equation 9) to express saturation - induced changes in metabolic acid-base status and thus total [CO_2_]. More correctly the Haldane BE coefficient varies with pH in a series of curves according to PCO_2_ and erythrocytic 2,3 diphosphoglycerate concentrations [8, 9].
5. As discussed above, compartmental ventilation in this model is a dependent variable. It also follows a log normal relationship with compartmental V/Q ratios, with its log SD and mean values fully defined by compartmental blood flow distribution parameters [Figures 1(s) and 2(s)]. In the West model distributions of blood flow and ventilation are imposed separately as independent variables and assessed separately by MIGET, with compartmental V/Q ratios themselves becoming dependent variables [1].
6. We opted for 20 gas exchanging compartments plus shunt for consistent data output. According to West, increasing model compartment numbers above 10 causes minimal changes in VO_2_, VCO_2_, PaO_2_ and PaCO_2_ [10].

Core Equations

1. Blood oxygen content (CbO_2_)

$$CbO_{2}=1.34.Hb\times S+0.003\times PO_{2}$$

2. Plasma CO_2_ content (CpCO_2_)

$$CpCO_{2}=2.226\times0.03.PCO_{2}.\left( 1+{10}^{pH-6.1} \right)$$

3. Blood CO_2_ content (CbCO_2_)

$$CbCO_{2}=CpCO_{2}\times\left[ 1-\frac{0.0289\times Hb}{\left( 3.352-0.456\times S \right).\left( 8.142-pH \right)} \right]$$

4. Plasma bicarbonate (Bicarb)

$$Bicarb=0.03\times PCO_{2}\times{10}^{pH-6.1}$$

5. Base excess (BE) [11]

$$BE=\left( 1-0.0143\times Hb \right)\times\left[ Bicarb-24.4+\left( 1.4276\times Hb+7.7 \right)\times\left( pH-7.4 \right) \right]$$

6. PCO_2_ from pH and BE

$$PCO_{2}=\frac{\left[ \frac{BE}{1-0.143\times Hb}-\left( 1.428\times Hb+7.7 \right)\times\left( pH-7.4 \right)+24.4 \right]}{0.03\times{10}^{pH-6.1}}$$

7. Actual P50 (P50act)

$$P50act=P50st\times{10}^{-0.48\times\left( pH-7.4 \right)}$$

8. Saturation (S) [5]

$$logitS=1.87+LN\left( \frac{PO_{2}}{1.955\times P50act} \right)+3.5\times TanH\left[ \frac{LN\frac{{PO}_{2}}{1.955\times P50act}}{3.5} \right]$$

$$S=\frac{EXP\left( logitS \right)}{1+EXP\left( logitS \right)}$$

9. Haldane correction (BEcorr)

$$\Delta S=S1-S2$$

$$\Delta BE=0.22\times Hb\times\Delta S$$

$$BEcorr=BE+\Delta BE$$

10. Venous admixture (VA)

$$PiO_{2}=FiO_{2}\times\left( BP-47 \right)$$

$$PAO_{2}=PiO_{2}-\frac{Pa{CO}_{2}}{R}$$

$$CcO_{2}=1.34.Hb\times SAO_{2}+0.003\times P{AO}_{2}$$

$$CvO_{2}=1.34.Hb\times SvO_{2}+0.003\times P{vO}_{2}$$

$$CaO_{2}=1.34.Hb\times SaO_{2}+0.003\times P{aO}_{2}$$

$$VA=100\times\frac{CcO_{2}-CaO_{2}}{CcO_{2}-CvO_{2}}$$

11. Oxygen delivery (DO_2_)

$$DO_{2}=CaO_{2}\times CO\times10$$

12. Mixed venous oxygen content (CvO_2_)

$$CvO_{2}=\frac{DO_{2}-VO_{2}}{10\times CO}$$

13. Mixed venous CO_2_ content (CvCO_2_)

$$CvCO_{2}=CaCO_{2}+0.1\times\frac{VCO_{2}}{CO}$$

14. Lung unit pre-equilibration

Assume Q = 100

Inspired gas volume (V)

$$V=\frac{v}{Q}\times100$$

1. Volume inspired nitrogen (VAN_2_)

$$VAN_{2}=V\times\left( 1-{FiO}_{2} \right)$$

VAN_2_ remains constant during equilibrations.

1. Inspired PO_2_ (PiO_2_)

$$PiO_{2}=\left( BP-47 \right)\times FiO_{2}$$

2. Inspired O_2_ content (O_2_v) (vol%)

$$O_{2}v=100\times\frac{PiO_{2}}{BP}$$

3. Lung unit total O_2_ content (O_2_goal)

$$O_{2}goal={CvO}_{2}+\frac{V}{Q}\times O_{2}v$$

ML: training and validation

Data

The dataset minus test-set consisted of 31097 rows. Each row contained the target values (Shunt, Log SD and Mean), plus 13 variables from the model input and output logs (Table 3, main article). Ten of these variables were analyzed for ‘Single–Point’ estimates of target values. These were: ‘CO_2_load’, ‘O_2_pull’, P50st, BE, Hb, FiO_2_, pH, PaCO_2_, PaO_2_, and VA. For ‘Two–Point’ estimates the variables DVA, Dsat and DPF were also included.

Data Pre-Processing

Exploratory data analysis was performed by generating scatter graphs of variables versus target attributes (Shunt, Log SD and Mean), and examining descriptive statistics, distributions, correlations and outliers using Pandas profiling and AutoViz libraries [12, 13]. After removal of duplicates and rows with missing values and sequestering 500 random rows for subsequent data validation, 30209 data rows remained for further evaluation.

Local outlier factor (LOF), a density-based detection method, was used to identify and remove outliers by applying 10^th^ nearest neighbour and Manhattan distance to the Single-Point variables. With hyperparameter tuning the contamination parameter predicting the proportion of anomalous data points was 0.02 [14].

The remaining 29064 rows were subjected to discretization (grouping continuous data into discrete buckets) using the feature engine DecisionTreeDiscretiser [15]. This was performed separately versus shunt for the CO_2_ - containing features (PaCO_2_ and CO2load) and O_2_ - containing features (FiO_2_, PaO_2_, and O2pull). A train-test split of 80:20 with 10-fold cross validation by negative mean square error generated separate models for discretization. Hyperparameter optimization was then undertaken using Grid Search with maximum Decision Tree depth of 1-4 and 5-fold cross-validation.

Feature engineering

Feature engineering focused on PF and R ratios (respectively PaO_2_ / FiO_2_ and CO2load / O2pull) and a linear combination of O2pull and CO2load. A quasi ‘Resting Energy Expenditure’ parameter was also calculated by substituting O2pull and CO2load for VO_2_ and VCO_2_ respectively in the abbreviated Weir equation [(3.94 x VO_2_) + (1.1 x VCO_2_)] [16].

Modelling

Modelling was performed by regression analysis optimizing mean absolute error and R^2^. Regression algorithms were accessed at one of six open-source Python Libraries (Table 1s)

Table 1 (s). Six open-source Python Libraries accessed for modelling

| Python Library | URL |
| --- | --- |
| Sklearn | https://scikit-learn.org/stable/supervised_learning.html#supervised-learning |
| LightGBM | https://lightgbm.readthedocs.io/en/latest/pythonapi/lightgbm.LGBMRegressor.html |
| Catboost | https://catboost.ai/en/docs/concepts/python-reference_catboostregressor |
| Cubist | https://github.com/pjaselin/Cubist |
| PyTorch | https://arxiv.org/pdf/1908.07442.pdf |
| EarthPy | https://contrib.scikit-learn.org/py-earth/content.html#bibliography |

Two configurations of regression algorithms were developed for the first based models and two different algorithms for the second stage models (meta-learners). The first based model combination was examined using 90:10 train-test split using 5-fold cross validation.

Initial algorithm screening was undertaken using the ‘lazy predict’ Python library and additional standalone deep learning (Tensorflow) and regression algorithms [17]. ‘Lazy predict’ is a wrapper library for comparing multiple regression (or classification) algorithms for modelling [18].

Algorithms were identified as ‘best performing’ based on R^2^ > 0.7. On this basis, screening identified K-nearest neighbours (KNN), light gradient boost machine (LGBM), extreme gradient boosting (XGB), random forest (RF), support vector machine (SVM), elastic net (EN), stochastic gradient descent regressor (SGD), decision tree (DT), classification and regression trees (CART), multi-layer perceptron (MLP), kernel ridge (KR), Bayesian ridge (BR), gradient boost tree (GBT), cat boost (CBR), TabNet [19] and multi-adaptive regression spline (MARS) as potential candidates.

First-Stage and Second Stage Regressors

A stacked regressor ensemble, formed by a linear combination of multiple prediction algorithms to improve overall prediction accuracy, provided the best results on both Single- and Two-Point data [20]. Final stacked regressor candidates were (KNN, LGBM, XGB, RF, SGD, EN, GBR, MARS, CBR) with second layer XGB meta-learner, and (KNN, CART, LGBM, XGB, SVM, RF, MLP, SGD, KR, EN, BR, GBR, CBR) with logistic regression meta-learner. Stacked regressors were built using the StackingCVRegressor library [21]. Pre-processing included standardization using Sklearn standard scaler.

Weighted Ensemble

A weighted ensemble using stacked regressor and Google’s TabNet was developed. TabNet is a deep learning model for tabular data which has outperformed leading tree - based models across a variety of benchmarks. It is more ‘explainable’ than boosted models and can be used without feature processing [19]. The Pytorch implementation of TabNet was used with a train-test-validation split of 80:10:10 using 1000 epochs, batch size 1024, patience of 50, and five- fold cross validation.

The weighted ensemble predictions (TabStack) were generated through a nonlinearly constrained gradient-based optimization sequential Least Squares programming (SLSQP) algorithm of the stacked regressor and TabNet predictions.

References

1. West JB, Wagner, PD. Pulmonary gas exchange. In: West JB, editor. Bioengineering Aspects of the Lung. New York: Marcel Dekker; 1977. p. 361-457.

2. Douglas AR, Jones NL, Reed JW. Calculation of whole blood CO2 content. J Appl Physiol (1985). 1988;65(1):473-7.

3. Kelman GR. Digital computer procedure for the conversion of PCO2 into blood CO2 content. Respir Physiol. 1967;3(1):111-5.

4. McHardy GJ. The relationship between the differences in pressure and content of carbon dioxide in arterial and venous blood. Clin Sci. 1967;32(2):299-309.

5. Siggaard-Andersen O, Siggaard-Andersen M, Fogh-Andersen N. The TANH-equation modified for the hemoglobin, oxygen, and carbon monoxide equilibrium. Scand J Clin Lab Invest Suppl. 1993;214:113-9.

6. Kelman GR. Digital computer subroutine for the conversion of oxygen tension into saturation. J Appl Physiol. 1966;21(4):1375-6.

7. Stainsby WN, Eitzman PD. Roles of CO2, O2, and acid in arteriovenous [H+] difference during muscle contractions. J Appl Physiol. 1988;65(4):1803-10.

8. Siggaard-Andersen O, Garby L. The Bohr effect and the Haldane effect. Scand J Clin Lab Invest. 1973;31(1):1-8.

9. Rees SE, Andreassen S. Mathematical models of oxygen and carbon dioxide storage and transport: the acid-base chemistry of blood. Crit Rev Biomed Eng. 2005;33(3):209-64.

10. West JB. Ventilation-perfusion inequality and overall gas exchange in computer models of the lung. Respir Physiol. 1969;7(1):88-110.

11. Siggaard-Andersen O. The Van Slyke equation. Scand J Clin Lab Invest Suppl. 1977;37(146):15-20.

12. Brugman S. Pandas Profiling 2021 [Available from: https://pandas-profiling.github.io/pandas-profiling/docs/master/rtd/.

13. Hiron R, Morena H. autoviz 0.1.35 2021 [Available from: https://pypi.org/project/autoviz/.

14. Xu Z, Kakde D, Chaudhuri A. Automatic Hyperparameter Tuning Method for Local Outlier Factor, with Applications to Anomaly Detection. 2019 [Available from: https://arxiv.org/abs/1902.00567.

15. Galli S. Feature-engine: A Python package for feature engineering for machine learning. . Journal of Open Source Software. 2021;6(65):3642.

16. Mehta NM, Smallwood CD, Joosten KF, Hulst JM, Tasker RC, Duggan CP. Accuracy of a simplified equation for energy expenditure based on bedside volumetric carbon dioxide elimination measurement--a two-center study. Clin Nutr. 2015;34(1):151-5.

17. M. Abadi AA, P. Barham, E. Brevdo, Z. Chen, C. Citro, G. S. Corrado, A. Davis, J. Dean, M. Devin, S. Ghemawat, I. J. Goodfellow, A. Harp, G. Irving, M. Isard, Y. Jia, R. Józefowicz, L. Kaiser, M. Kudlur, J. Levenberg, D. Mane, R. Monga, S. Moore, D. G. Murray, C. Olah, M. Schuster, J. Shlens, B. Steiner, I. Sutskever, K. Talwar, P. A. Tucker, V. Vanhoucke, V. Vasudevan, F. B. Viégas, O. Vinyals, P. Warden, M. Wattenberg, M. Wicke, Y. Yu, and X. Zheng. TensorFlow: Large-Scale Machine Learning on Heterogeneous Systems. arxivorg/abs/160304467. 2016;Software available from tensorflow.org.

18. Pandala S. Lazy Predict 2020 [Available from: https://lazypredict.readthedocs.io/en/latest/readme.html.

19. Arik S, Pfister T. TabNet: Attentive Interpretable Tabular Learning. arXiv. 2020;1908.07442.

20. Wolpert D. Stacked generalization. Neural Netw. 1992;5(2):241–59.

21. Raschka S. StackingCVRegressor 2020 [cited 2022 18th January]. Available from: http://rasbt.github.io/mlxtend/user_guide/regressor/StackingCVRegressor/.
